# Differential Microstrip Sensor for Complex Permittivity Characterization of Organic Fluid Mixtures

**DOI:** 10.3390/s21237865

**Published:** 2021-11-26

**Authors:** Amer Abbood al-Behadili, Iulia Andreea Mocanu, Teodor Mihai Petrescu, Taha A. Elwi

**Affiliations:** 1 Department of Electrical Engineering, College of Engineering, Mustansiriyah University, Baghdad 00964, Iraq; amer_osman@uomustansiriyah.edu.iq; 2 Department of Telecommunication, Telecommunications and Information Technology, Faculty of Electronics, University Politehnica of Bucharest, 060042 Bucharest, Romania; teodor.petrescu@upb.ro; 3 Communication Engineering Department, Al-Ma’moon University College, Baghdad 1104, Iraq; taelwi82@gmail.com; 4 Electrical and Computer Engineering Campus, New York Institute of Technology, Long Island City, NY 11568, USA

**Keywords:** differential microstrip sensor, urine sensor, complex permittivity, open-stub resonator

## Abstract

A microstrip highly sensitive differential sensor for complex permittivity characterization of urine samples was designed, fabricated and tested. The sensing area contains two pairs of open-stub resonators, and the working frequency of the unloaded sensor is 1.25 GHz. The sensor is easily implemented on an affordable substrate FR-4 Epoxy with a thickness of 1.6 mm. A Teflon beaker is mounted on the sensor without affecting the measurements. Numerically, liquid mixtures of water and urine at different percentages were introduced to the proposed sensor to evaluate the frequency variation. The percentage of water content in the mixture varied from 0% (100% urine) to 100% (0% urine) with a step of 3.226%, thus giving 32 data groups of the simulated results. Experimentally, the mixtures of: 0% urine (100% water), 20% urine (80% water), 33% urine (66% water), 50% urine (50% water), 66% urine (33% water), and 100% urine (0% water) were considered for validation. The complex permittivity of the considered samples was evaluated using a nonlinear least square curve fitting in MATLAB in order to realize a sensing sensitivity of about 3%.

## 1. Introduction

Resonating sensors are widely used in different applications such as: solid dielectric characterization [1,2,3], biomedical application [4,5], permittivity measurements for liquid mixtures [6,7,8], or even characterization of soil water content [9,10]. Generally, the most used are planar sensors due to their low cost, low profile, easy fabrication, high precision, robustness, and compact size [11]. The sensing principle of such sensors is based on detecting the change in the resonant frequency when placing a sample over the resonating surface [11].

The sample can be both a solid material and a liquid. Particular attention has been given to sensors for measuring the dielectric properties of microfluids. Usually, a mixture of water and inorganic fluids is used to determine these properties and few papers address this aspect when it comes to organic fluids.

One of the important organic fluids of human biological liquids is urine [11]. Urine is a liquid waste of the body consisting of water, inorganic salts, and organic compounds [3]. Urine color, which depends on the proportion between metabolites and water, can be used to detect a person’s hydration state and early dehydration problems [12]. Thus, a urine color chart was developed by Armstrong in 1994 for hydration assessment [13]. In particular, unconscious and elderly patients need their hydration state monitored. Water balance in the human body is a key indicator for good functioning of different metabolic activities [14]. In particular, the hydration state of a person is influencing blood pressure, heart rate, body temperature, etc. Thus, it is very important to have accurate measurements about this state. The hydration assessment techniques for the body involve urinary, hematologic, whole-body, and sensory measurements [15]. Determining the level of hydration when analyzing urine is one of the most efficient, easy, and least invasive methods, so sensors capable of doing this have been investigated often.

Differential sensors are mostly used because they are robust against variations in ambient factors [16,17,18,19,20,21,22]. Differential sensors are typically implemented by means of two sensing elements, e.g., two loaded transmission lines. The sensing principle practically relates to symmetry. Under perfect symmetry, the structure exhibits a single transmission zero frequency. When loading the sensor with samples on one side, the symmetry is interrupted, and two resonant frequencies appear [19]. One limitation of these frequency-splitting sensors may be caused by the possible coupling between resonant elements, which is unavoidable when these elements are too close [22]. To avoid this phenomenon and to obtain the advantages of differential sensors, in this paper a sensor consisting of two identical parts is considered. One part is made of a Wilkinson power divider and two transmission lines loaded with a pair of open-stub resonators each. The two sensing parts are placed far one from another not to have couplings between the elements. The sensing area is covered by a Teflon beaker without affecting the sensitivity of the sensor. The beaker is used to pour liquids in it and to make precise measurements.

## 2. Sensor’s Design

### 2.1. Resonant Structures for the Sensor’s Design

In the literature there are two types of substrates for designing microfluidic substrate: rigid and flexible [23]. The flexible ones, such as Polydimethylsiloxane (PDMS), paper, and polyimide have the main advantage of being compatible with additive manufacturing techniques, but some of the drawbacks referring to their usage are surface treatment, incompatibility with ink solutions (chemicals), and sensitivity to thermal sintering [23]. On the other hand, the rigid substrates have the advantage of having constant dielectric properties (ε_r_ and tan δ) even at different temperatures and frequencies, low-losses, and are affordable [23]. This is the reason why, for our design a rigid substrate is chosen. The sensor is designed in microstrip technology on an affordable substrate, FR-4 Epoxy with relative permittivity *ε*_r_ = 4.4, thickness *h* = 1.6 mm, and loss tangent tan *δ* = 0.02. For technological reasons, the width of the microstrip transmission lines must be greater than 0.5 mm. The working frequency of the sensor is then set to 1.245 GHz for fulfilling the technological restrictions imposed.

**Figure 1:**
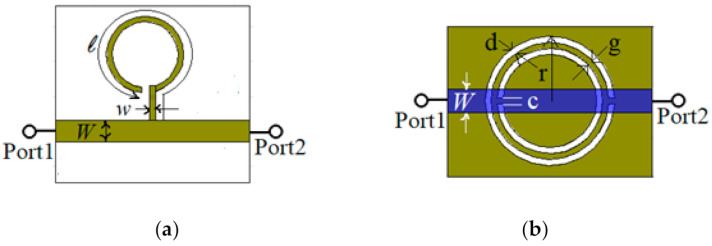
Microstrip transmission line loaded with: (**a**) a λg/4 open stub resonator with physical dimensions; (**b**) a CSRR etched in the ground with physical dimensions.

To start, two microstrip lines are designed and analyzed through simulations: one loaded with a λ_g_/4 open stub resonator, as depicted in Figure 1a, and another one loaded with a CSRR etched in the ground as depicted in Figure 1b. The guide wavelength, λ_g_ is the one corresponding to the FR-4 Epoxy substrate at the operating frequency of 1.245 GHz. The length of the open stub resonator is *ℓ* = 53.45 mm. The width of the microstrip open resonator is *w* = 0.8 mm, while the width of the loaded transmission line is *W* = 3.083 mm, which corresponds to the characteristic impedance of 50 Ω for the access transmission line at the operating frequency. The length of the access transmission line is set to 28.6 mm.

The CSRR loaded line is designed to work at the same resonant frequency as the open stub resonator, so the width of the loaded transmission line is *W* = 3.083 mm, the length is set to 28.6 mm, the width of the rings and the distance between them is d = g = c = 0.8 mm, and the radius of the exterior ring is set to r = 8.325 mm.

**Figure 2:**
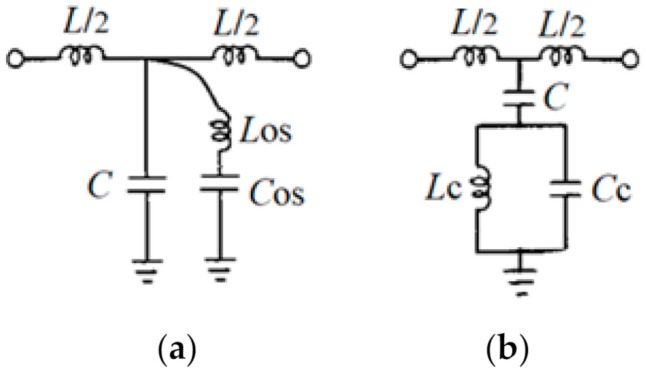
Equivalent circuit for the: (**a**) λg/4 open stub resonator; (**b**) CSRR etched in the ground.

The equivalent circuits of the two resonating structures are depicted in Figure 2a,b. For the resonating structure in Figure 1a, the feeding line between ports 1 and 2 is modeled by an inductance, *L* and a capacitance *C*, while the open stub is modeled by an inductance *L*_os_ and a capacitance *C*_os_. For the resonating structure in Figure 1b, the feeding line between ports 1 and 2 is modeled by the inductance *L* and the capacitance *C*, while the CSRR is modeled by an inductance and capacitance *L*_c_ and *C*_c_, respectively [24].

The characteristic impedance of the transmission line between ports 1 and 2 is *Z*_0_ = 50 Ω and the substrate is FR-4 Epoxy, so the values for the inductance *L* = 8.9546 nH and, the capacitance *C* = 1.6798 pF are the same for both equivalent circuits. Considering the geometrical dimensions of the two resonating structures and the same substrate, the values for the other lumped elements from the equivalent circuit are extracted [17]: *L_c_* = 6.0958 nH, *C_c_* = 2.085 pF, *C_os_* = 1.69 pF, and *L_os_* = 9.528 nH.

In this case, the resonant frequency of the equivalent circuit in Figure 2a can be written as:

$$f_{os}=\frac{1}{2\pi \sqrt{L_{os}C_{os}}}=1.248\;\mathrm{GHz}$$

for the equivalent circuit in Figure 2b:

$$f_{o}=\frac{1}{2\pi \sqrt{L_{c}(C+C_{c})}}=1.25\;\mathrm{GHz}$$

The resonant frequency obtained using the equivalent circuits is equal to the one imposed by design.

**Figure 3:**
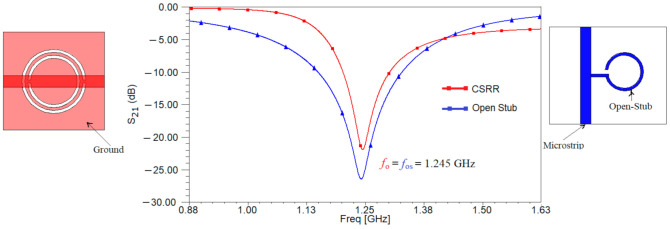
Transfer parameter, S_21_ (dB) for the microstrip line loaded with a CSRR etched in the ground, respectively with an open stub.

The next step in the design of the sensor is to compare the performances of the two resonant structures designed in Figure 1 and decide which one is best suited for our application. Using the full-wave simulator High Frequency Structure Simulator (HFSS) both structures are analyzed. The results of the simulation for the transfer characteristic are depicted in Figure 3. The resonant frequency is the same for both resonant structures, proving that the designed is correct. Moreover, the resonant frequency is equal to the ones determined using the equivalent circuits from Figure 2.

Analyzing the results in Figure 3, it can be seen that the microstrip line loaded with an open stub has a value of 26 dB for parameter S_21_ rather than only 22 dB as in the case of the microstrip line loaded with a CSRR etched in the ground.

**Figure 4:**
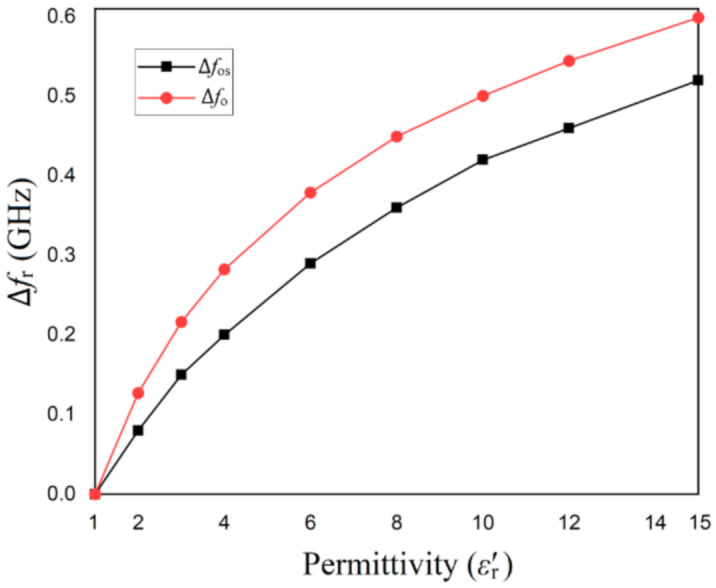
The resonant frequency shift Δ*f_r_* for the microstrip line loaded with an open-stub resonator and a CSRR etched on the ground, respectively when changing the real part of the relative permittivity of the surrounding medium.

The sensitivity of the resonant structures is investigated by placing the two structures in a box and modifying the medium’s characteristics inside the box. The resonant frequency of each structure when the box is filled with vacuum is determined by simulation in HFSS and is considered the reference resonant frequency for each structure. Then, the box is filled with different other media and the resonant frequency is determined by simulation. The difference between the new resonant frequency and the reference one is computed and plotted as a function of the real part of the permittivity of the medium, as depicted in Figure 4.

The results of the simulation in Figure 4 show that the CSRR structure offers a higher resonance frequency shift than open stub structure when changing the real part of the permittivity. Starting from a value of around 4 for the real part of the permittivity, the difference between the two structures remains the same in terms of sensitivity.

On the other hand, the influence of medium’s imaginary part of the permittivity over the frequency shift must be investigated for both structures. For this, the loss tangent of the medium is changed and the amplitude of the transfer parameter, S_21_ is determined through simulation. Based on these values, the quality factor for each resonant structure is determined as a function of the loss tangent of the medium.

**Figure 5:**
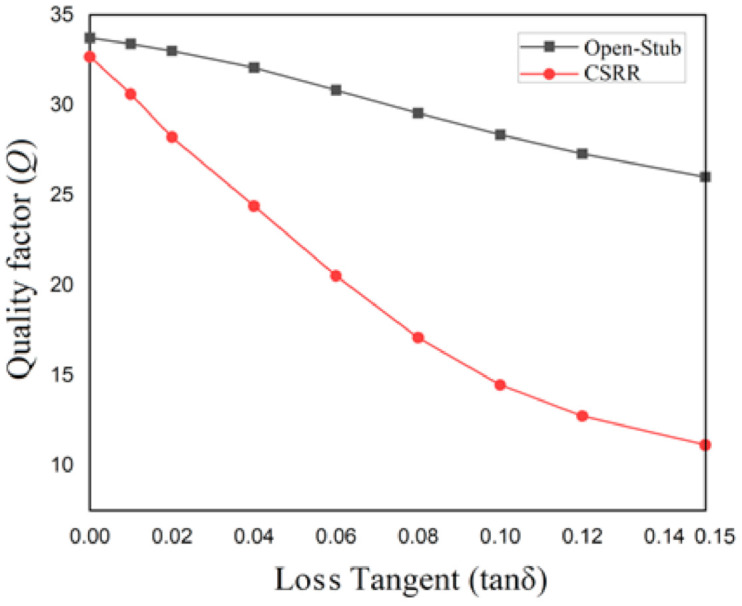
Quality factor for each resonant structure when changing the loss tangent of the medium inside the box.

The quality factor for general resonators, *Q* can be written [23]:

(1) $$Q=\frac{f_{r}}{\Delta f}$$

where *f_r_* is the resonant frequency and ∆*f* represents the relative 3 dB bandwidth of the resonator’s frequency response. The results are given in Figure 5.

From Figure 5, one can notice that the quality factor of the CSRR structure decreases drastically with the increase of the medium’s loss tangent. This means, that the open stub structure is better suited to applications which use samples with a large loss tangent, as urine, for example. This is the reason for which the sensor will be implemented using the open-stub resonator.

### 2.2. Sensor’s Layout

After considering the best suited resonant structures for our application, the next step is to design the whole sensor. It will be a differential one, consisting of two identical Wilkinson power dividers and two pairs of microstrip transmission lines, each of them loaded with two open stub resonators as designed in the previous Section. Additionally, a beaker made of Teflon is perfectly attached to the surface of the two open stub resonators to avoid any measurement discrepancy because of the air gap effects between the beaker and the sensor. The beaker is filled with liquids that will be considered samples under test (SUT).

**Figure 6:**
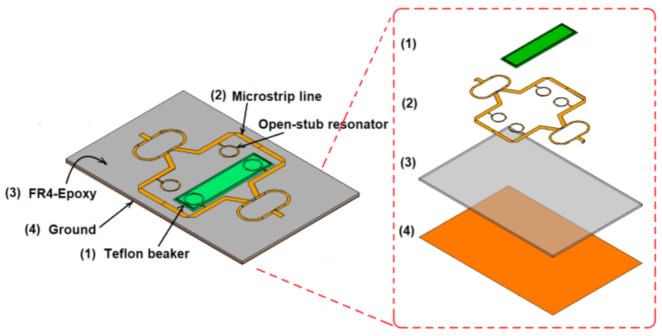
The layout of the sensor, including an exploded view drawing of each layer.

The layout of the sensor is given in Figure 6.

As it can be seen in Figure 6, the power applied at one of the two ports is divided equally by the Wilkinson power divider and then transmitted to the first pair of resonant structures. As the sensor is symmetrically designed, once a sample is placed over one pair of resonant structures, it will affect the symmetry of the whole structure and this will be seen as a shift in the reference resonant frequency. Additionally, because of samples that possesses different electrical parameters, two resonant frequencies will appear, each given by one pair of the resonant structures.

**Figure 7:**
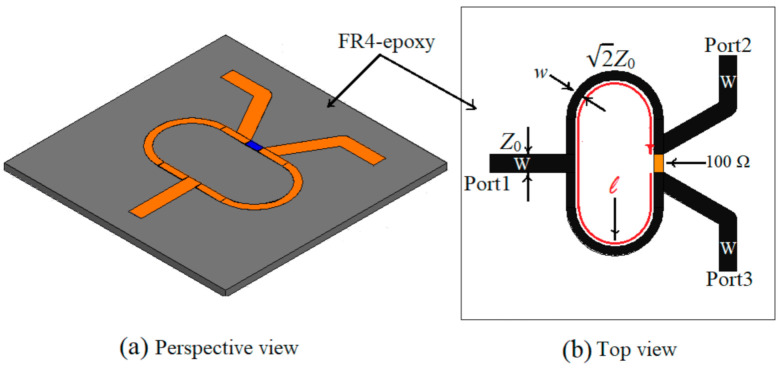
Wilkinson power divider: (**a**) perspective view; (**b**) top view.

As described previously, the microstrip lines loaded with open-stub resonators are the ones designed in Section 2.1, so the next step is to design the Wilkinson power divider. The substrate is the same, FR-4 Epoxy with a thickness of 1.6 mm and the central frequency is 1.245 GHz. In this case, the electrical and physical dimensions for the Wilkinson power divider as well as the layout of the divider are given in Figure 7 and Table 1.

**Figure 8:**
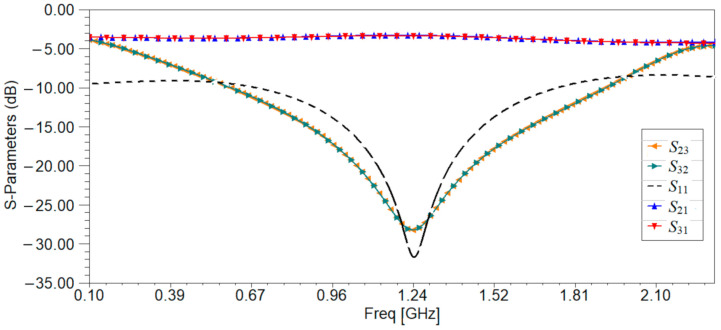
The scattering parameters of the Wilkinson power divider.

The frequency behavior of the Wilkinson power divider is simulated, and the results are given in Figure 8.

Figure 8 shows that the Wilkinson power divider works at the frequency of 1.245 GHz with a return loss better than 30 dB, an isolation loss of 27 dB, and an insertion loss of 3.25 dB, so it can be successfully used for the sensor’s design.

**Figure 9:**
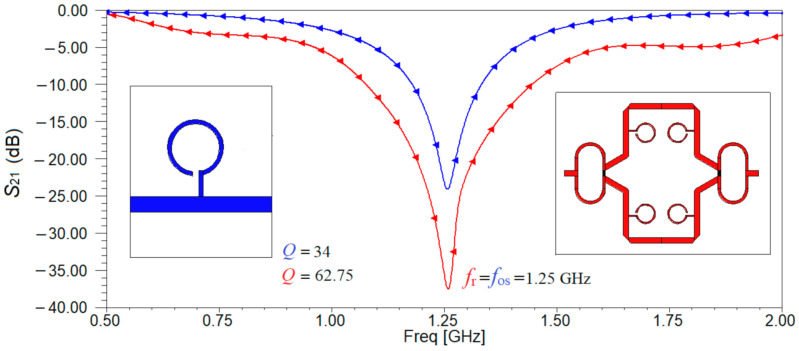
The transmission coefficient for the two resonant structures: blue line for the microstrip transmission line loaded with open-stub resonator, red line for the sensor. The resonance frequencies and the quality factors are also given.

Next, a comparison between the sensor’s transmission characteristic and the one of the microstrip transmission line loaded with an open stub resonator is carried and the results of the simulation are depicted in Figure 9. It can be seen that in the case of the sensor, a minimum of the transmission characteristic is obtained at a frequency very close to the imposed one which is now 1.25 GHz. Additionally, we can determine the quality factors corresponding to each resonating structures as being equal to 34 and to 62.75, respectively using Relation (1).

Analyzing the results in Figure 9, it can be seen that by adding pairs of resonators, the quality factor increases by 84.56% than in the case of one resonator and the fact that the transmission characteristic becomes sharper at the resonant frequency, providing a better accuracy to characterize the complex permittivity of the samples.

**Figure 10:**
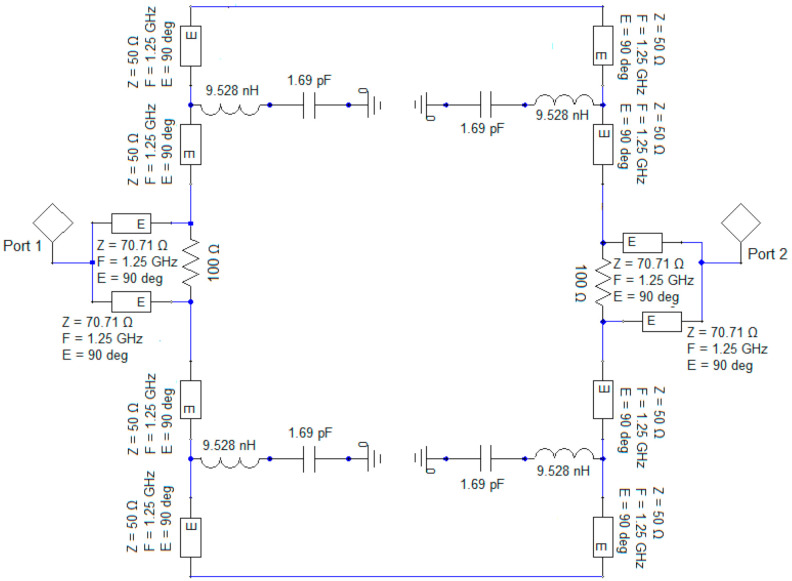
The equivalent lumped circuit of the proposed sensor.

For a better understanding of the operational principal of the sensor, the equivalent circuit model is depicted in Figure 10. The resonant structure is replaced by its equivalent lumped circuit from Figure 2a and the Wilkinson power divider is made of two identical transmission lines each having an electrical length E = 90° and a characteristic impedance 70.71 Ω, isolated by a resistor of resistance *R* = 100 Ω. The value for the resistance of the resistor used to design the power divider is obtained by imposing that the two output ports of the Wilkinson divider are matched. The analysis of the three-port power divider is done using the even-odd excitation principle. Based on these two considerations, the value of the resistor placed in the Wilkinson power divider to isolate the output ports is determined, *R* = 2*Z*_0_ = 100 Ω. The transmission lines loading the resonant structure have the characteristic impedance equal to 50 Ω and an overall electrical length of 360° in order to not introduce additional phase shifts.

Practically, the power at port 1 is divided equally by the Wilkinson power divider and each pair of resonating structure receives equal power. Due to the symmetry of the sensor, the same power arrives at the outputs of the second Wilkinson power divider and is summed at port 2. In fact, at port 1 we have connected the input of a Wilkinson power divider and at port 2, we have connected the output of an ideal Wilkinson power combiner. When adding liquid for test in the Teflon beaker placed above one of the sensing areas, the symmetry is broken, and a second resonant frequency appears. This is used for measuring the frequency shift and determine further on the electrical properties of the liquid sample.

**Figure 11:**
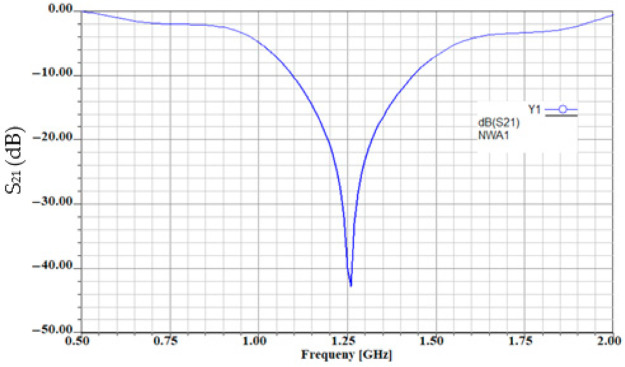
The transmission coefficient of the equivalent lumped circuit for the sensor.

The transfer characteristic of the equivalent circuit is obtained in Ansoft Designer by simulation and is given in Figure 11.

Comparing the results in Figure 9 and Figure 11, it can be noticed that for both circuit analysis and electromagnetic simulation, the resonant frequency remains 1.25 GHz and the response in frequency of the transmission coefficient is identical. The losses considered by the electromagnetic simulator can be seen in the value of parameter S_21_ which is only −38 dB compared to −41.3 dB.

### 2.3. Sensor’s Performance through Simulations

**Figure 12:**
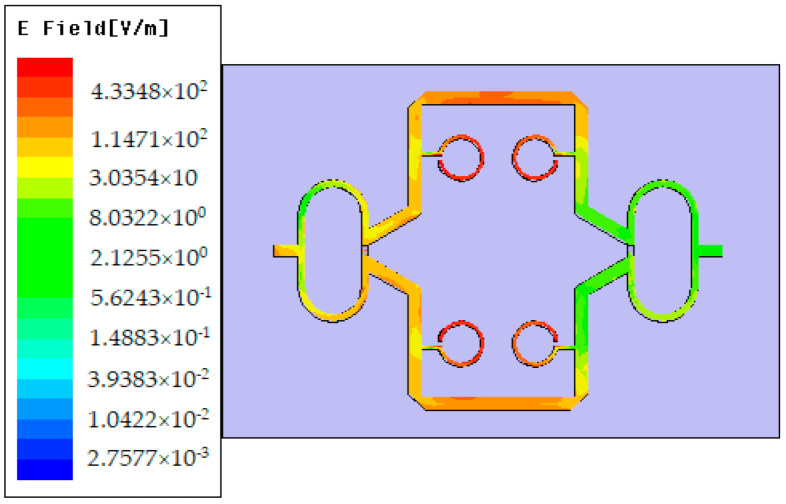
Intensity of the electric field at the resonant frequency of 1.25 GHz.

To verify the sensor’s performance, the distribution of the electric field at the resonant frequency of 1.25 GHz is analyzed. The results are given in Figure 12. It can be noticed that a high concentration of the intensity of the electric field can be found in the open-stub resonators, so the SUT must be placed over them.

**Figure 13:**
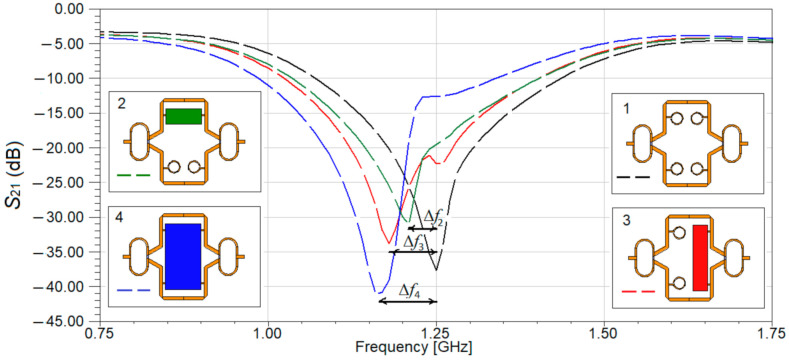
Simulated transmission characteristic, S_21_ of the proposed sensor for different positions of the sample: 1. no beaker, 2. longitudinal position of the beaker, 3. transversal position of the beaker, 4. beaker positioned on the whole sensing area.

Next, we investigate the best position of the sample over the resonant structures. Three cases are considered and the SUT is FR-4 epoxy with the same thickness. As a reference for the frequency shift, Δ*f*, the resonant frequency of the sensor without a sample is considered. The results of the simulation are given in Figure 13.

The reference for resonant frequency in the first case, when no SUT is mounted over the proposed sensor, is 1.25 GHz. The simulated frequency shifts for the second, third and fourth configurations depicted in Figure 13 are Δ*f*_2_ = 40 MHz, Δ*f*_3_ = 80 MHz, and Δ*f*_4_ = 100 MHz, respectively. The values for the S_21_ parameters in the second, third and fourth case, measured at the resonant frequency are −31 dB, −33 dB, and −41 dB, respectively. We must remember that the sensor is differential, so the symmetry must be broken when placing the SUT, so even if a better frequency shift is obtained for the fourth case, the sensor is not a differential one anymore [23]. Thus, taking this into account and considering the performances of the sensor for both the frequency shift and the amplitude of the S_21_ parameter at the resonant frequency, the third configuration offers the best results.

Next, we want to investigate the influence of the beaker over the performances of the sensor. Ideally, adding the beaker should have no influence over the performances, allowing the lines of electric field to go through the liquid and the sensing area. In addition to that, it should be able to keep the liquid distributed uniformly over the sensing area, increasing the sensing precision. For this reason, a Teflon beaker is chosen, as Teflon is a bio suitable material; is solid and soft enough to process for microfluidic devices; has very small losses (loss tangent of 0.001); and a small permittivity (ε_r_ = 2.1), which does not affect the behavior of the sensor. In addition, we mounted a Teflon beaker instead of etching a microfluidic channel made of PDMS because it requires less technological precision and to avoid any fabrication tolerance, but still maintained the sensing performances as is demonstrated through simulations, as shown in Figure 11. The proposed Teflon beaker width is 13 mm, the length is 60 mm, the height is 1 mm, and the base thickness is 0.1 mm. These dimensions provide a volume of 0.78 mm^3^. If we consider the density of water 997 kg/m^3^ and the one of urine from a healthy person between 1015–1022 kg/m^3^, this means that the liquid samples needed to make measurements have to be around 0.78 mL. On the other hand, urine is a biological liquid that is used for numerous tests, and it can be provided in large quantities, not like sweat for example, so the capacity of the beaker is not an issue.

**Figure 14:**
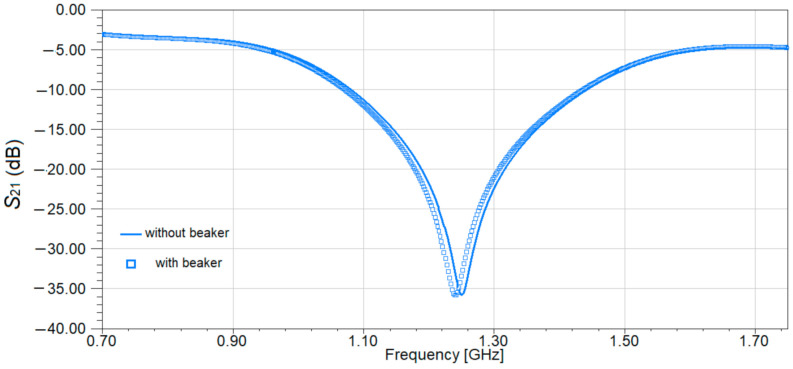
Transmission characteristic of the sensor without and with a beaker on top.

The Teflon beaker is located on the proposed sensor’s top surface, covering one pair of open stub resonators. To make sure that the beaker does not affect the sensor’s performance, a simulation of S_21_ parameter was carried in HFSS for two cases: the sensor with and without a beaker on top. The results of the simulation are given in Figure 14.

**Figure 15:**
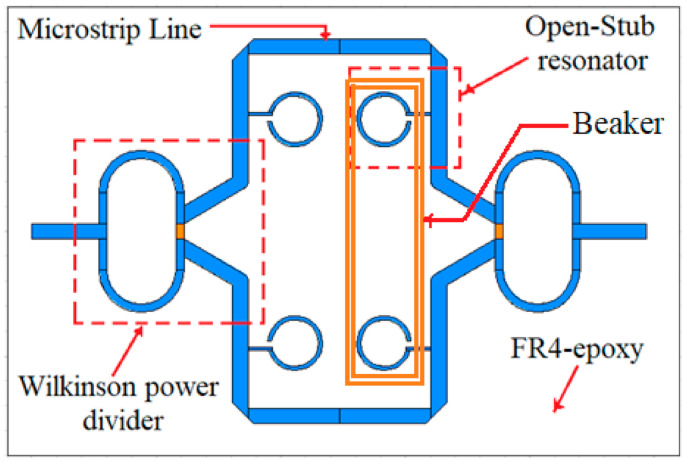
Top view of the sensor with beaker attached.

Analyzing the data in Figure 14, it can be observed that the influence of the beaker is minimal compared to the overall characteristic, due to the very small values for the dielectric constant and loss tangent for Teflon. The results in Figure 14 compared to the ones in [18] show the importance of the material used for beaker or the microfluidic channels. In [18] PDMS was used to create the channels and the results in Figure 15 [18] show a dramatic decrease of the transmission coefficient S_21_ from almost −50 dB without channels to −20 dB with channels. In our case, when using a Teflon beaker, the value of the S_21_ parameter remains almost constant at a value of −35 dB, proving that Teflon is a good choice for this application. In these conditions, the sensor proposed in this study will have the design as the one in Figure 15.

The sensor presented in Figure 15, is a differential sensor as it is made of two identical sensing areas: one Wilkinson power divider/combiner and a pair of transmission lines loaded with two open-stub resonators. As the Teflon beaker has no influence on the frequency response of the sensor, as proved in Figure 14, the sensing principle is similar to a differential sensor [23]: by loading the sensor with liquids under test (LUT) in the beaker, the symmetry is broken, and another resonant frequency appears. The reference will be considered the case when the sensor is loaded with pure water. This behavior will be proven by the results of both simulations and measurements for different organic mixtures in the next sections.

**Figure 16:**
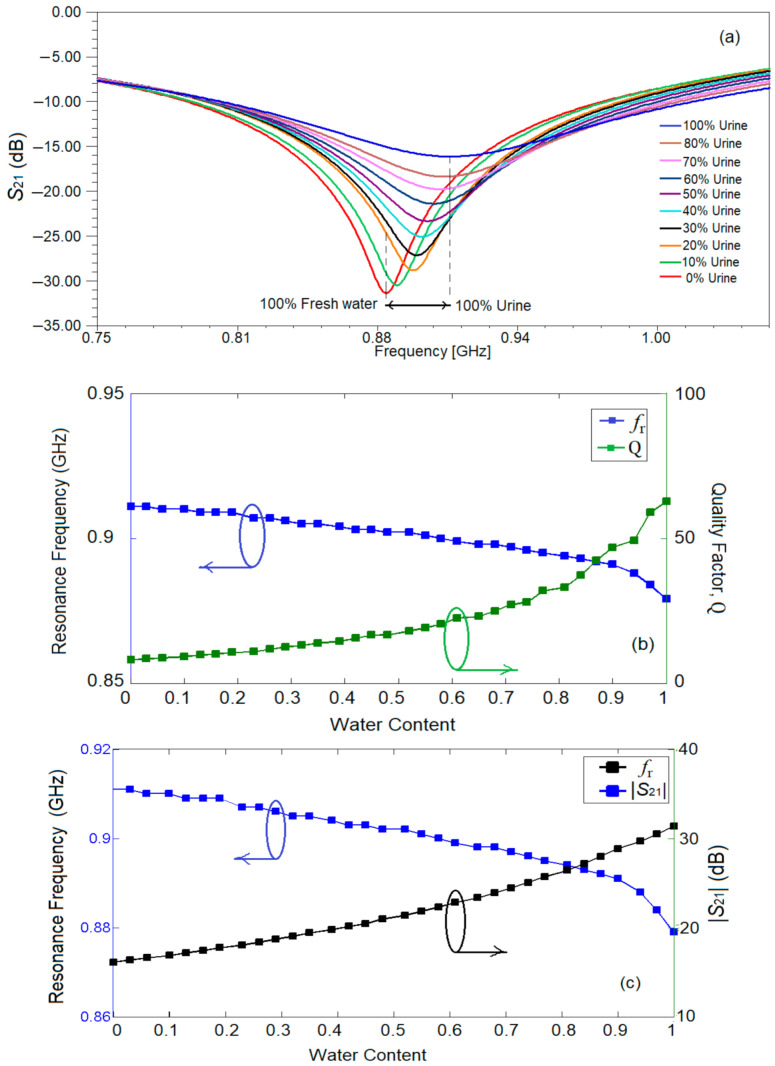

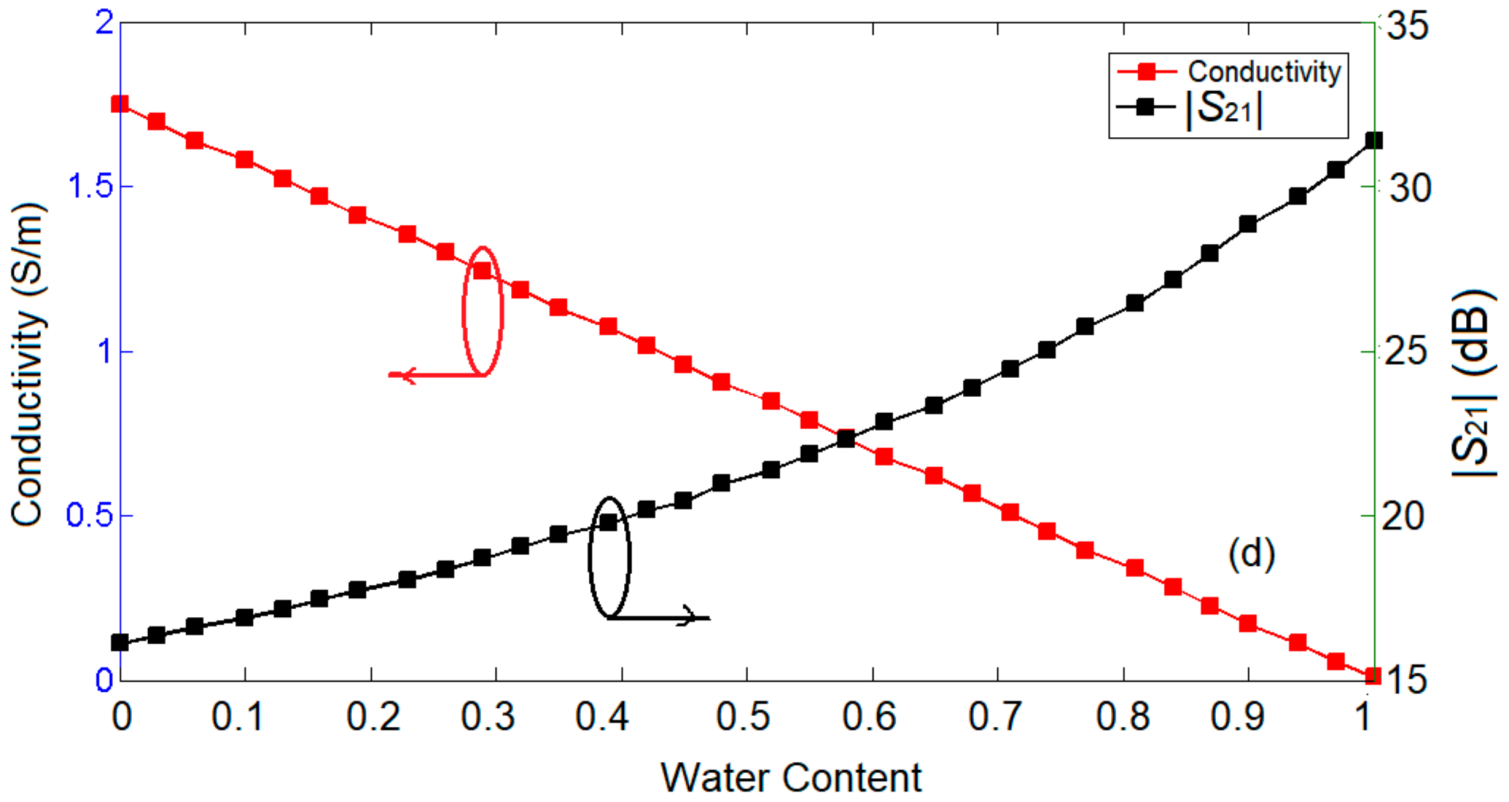
(**a**) Insertion loss for 10 ratios of water–urine mixture; (**b**) quality factor and resonance frequency for 32 ratios of water–urine mixture; (**c**) insertion loss and resonance frequency for 32 ratios of water–urine mixture; (**d**) conductivity and insertion loss for 32 ratios of water–urine mixture.

Next, liquid mixtures of water and urine in different percentages are used to simulate the sensor’s frequency behavior. The percentage of water content in the mixture is varied from 0% (100% urine) to 100% (0% urine) with a step of 3.226% providing 32 data groups of the simulated results. The values for the relative permittivity for water and for urine are 50 and 81, respectively, and for conductivity the values are 0.01 S/m and 1.75 S/m, respectively [25]. The simulation results for the transmission characteristic of the sensor for some of the cases is given in Figure 16a. All cases are considered for further analytical computations and for determining the quality factor, the resonance frequency and conductivity, in Figure 16b–d. The quality factor was determined using Relation (1) and the data provided by simulation for the insertion loss and conductivity.

Analyzing the data in Figure 16a,b, it can be observed that the resonant frequency increases with the increase of urine concentration in the mixture and the quality factor decreases with the increase of water concentration in the mixture. The resonance frequency decreases from 0.908 GHz when the mixture consists of 100% urine to 0.879 GHz when the mixture consists of 100% water. Additionally, from Figure 16a one can notice that the resonance peak is not so well emphasized when the quantity of urine increases.

Analyzing the results in Figure 16c, it can be seen that the slope of insertion loss variation is larger than the one of resonant frequency. The maximum variance in magnitude for the insertion loss reaches up to 15.272 dB (−31.4 dB for pure water to −16.13 dB for pure urine), while the maximum variance in resonant frequency is 32 MHz (0.879 GHz for pure water to 0.911GHz for pure urine).

The conductivity analysis given in Figure 16d shows a larger variation than the ones for the parameters in Figure 16b,c, so the parameters in Figure 16d will be used for measuring the complex permittivity of the urine–water mixture.

Nevertheless, these results conclude that the electrical parameters of the mixture are highly influenced by the content of salt and water in the urine.

## 3. Results

The sensor proposed in Figure 15 is now implemented and measured. The substrate used is FR-4 (relative permittivity *ε*_r_ = 4.4 and the dissipation factor, tan δ, is approximately 0.02), with a thickness of 1.6 mm and cooper metallization electrodeposited on both sides of the substrate, with a thickness of 18 µm.

**Figure 17:**
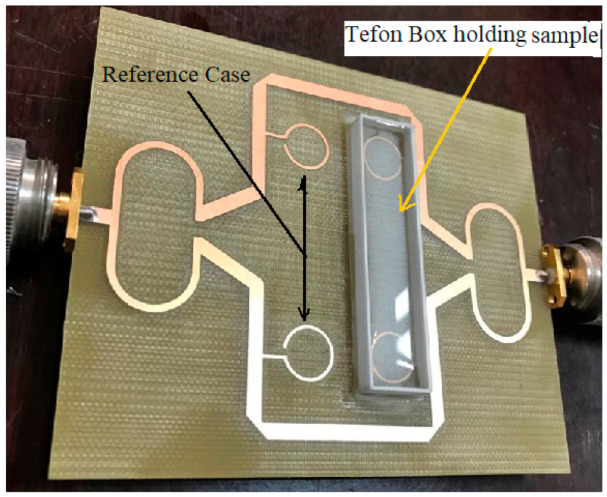
Photograph of the fabricated sensor for measuring different urine samples.

The SMA (SubMiniature version A) connecters, which are classical semi-precision coaxial RF connectors used as interface for coaxial cables with screw-type coupling mechanism are mounted on the structure using mechanical welding. The SMA has a 50 Ω characteristic impedance and is designed to work in the range 0–18 GHz, fully matched with the necessities of the current sensing structure. The beaker is made of Teflon and carefully glued to the sensing area, making sure no air gap exists. The manufactured sensor is presented in Figure 17.

The measurement setup consists of the sensor connected to the Agilent E5071C, Agilent Technologies (Keysight Technologies), USA (9 kHz to 6.5 GHz) network analyzer through 50 Ω cables. Before starting the measurements, a short-open-load-through (SOLT) calibration was carried out using the Agilent calibration Kit. The number of sweep points is chosen 1601.

The resonant frequency measured for the empty sensor was 1.25 GHz as in simulations, verifying that the sensor has been implemented correctly.

**Figure 18:**
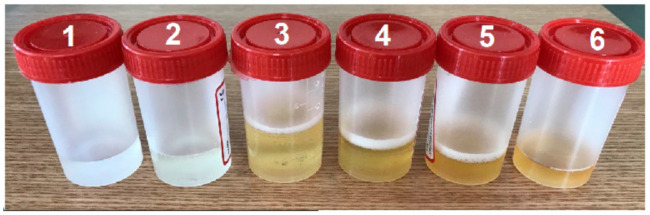
Different ratios urine-water mixtures used for measurements.

A set of samples under test is selected and used for measurements. The pure urine sample ($\varepsilon_{r}$ = 50, 1.75 S/m) is used only for obtaining calibration curves which are then used to characterize the urine-water mixture samples. Practically, healthy male urine is combined with water in different ratios and six samples are obtained as explained in Table 2 and depicted in Figure 18.

**Figure 19:**
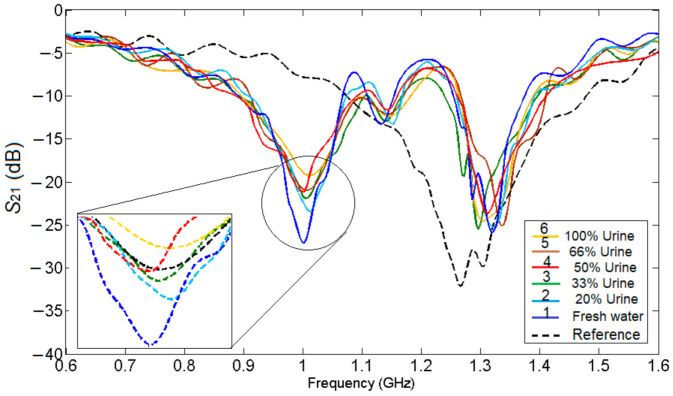
Measurement results for the samples presented in Figure 18, where the reference is represented by the unloaded sensor.

For each measurement, the sensor is placed on a rough, stable surface and the SUT is carefully placed to cover the whole sensing area, making sure no pellicular effect exists. To reduce the effect of impurities and of humidity from previous tests, the beaker is washed thoroughly, then rinsed with water and dried by cotton brushes. Finally, the next urine sample is dropped in the beaker. Then, using the Agilent E5071C network analyzer, the magnitude of S_21_ parameter is measured. The measurements have been repeated four times in the same environmental conditions. The results of the measurements are given in Figure 19.

Analyzing the results in Figure 19, it can be seen that the differential behavior of the sensor, meaning when it is not loaded, only one resonant frequency appears and once mixtures are added into the beaker, the symmetry is broken, and two resonant frequencies appear. One advantage of using a Teflon beaker instead of microfluidic channels is that no synchronization is required when filling the beaker.

The measurement results in Figure 19 show that the maximum value for the transmission parameter, S_21_ is obtained at −26.93 dB for 100% water and starts to decrease once urine is added to the mixture, reaching a minimum at −19.32 dB when the sample contains 100% urine, so we can conclude that the sensor works properly.

The largest variance introduced by the insertion loss is 7.61 dB, this is about half of the difference obtained by simulation due to using a water-free urine sample. As a result, the morning urine sample was calibrated to the new condition starting at 50% of the simulation of water content in mixture.

The calibration of water content is derived as:

(2) $$W_{sam}^{\prime }=\frac{W_{sam}+100}{2}$$

where $W_{sam}^{\prime }$ represent calibrated water content and $W_{sam}$ is water content ratio according to Table 2.

**Figure 20:**
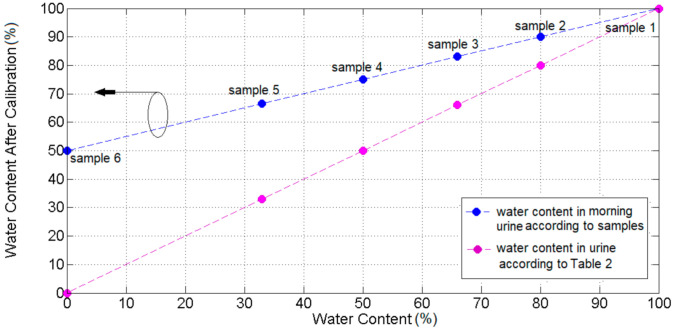
The difference between water content ratios in Table 2 and that obtained using Equation (2).

Calibrated ratios of water content in each sample corresponding to that in Table 2 are depicted in both Figure 20 as well as Table 3.

Regarding the resonant frequency for all the samples, it is clear that frequency shifts occur, and the values are different depending on the quantity of urine that was been added to the mixture. The data is synthesized in Table 3.

Further on, to obtain the values for the complex permittivity of the urine-water mixture, a nonlinear least square curve fitting in MATLAB is used to derive an equation describing the relation between variations of the resonance frequency and peak attenuation as a function of the complex permittivity variations [26,27]:

(3) $$\left[\begin{array}{c} \Delta f\\ \Delta \left|S_{21}\right| \end{array}\right]=\left[\begin{array}{cc} m_{11} & m_{12}\\ m_{21} & m_{22} \end{array}\right]\cdot \left[\begin{array}{c} {\Delta \varepsilon \prime }_{\mathrm{sam}}\\ {\Delta \varepsilon \prime \prime }_{\mathrm{sam}} \end{array}\right]$$

where the following notations have been used: Δε′_sam_ = ε′_sam_ – ε′_ref_, Δε″_sam_ = ε″_sam_ – ε″_ref_, Δ*f*_sam_ = Δ*f*_sam_ − Δ*f*_ref_, and Δ|S_21_| = Δ|S_21_|_sam_ − Δ|S_21_|_ref_, with subscript “sam” for the sample and “ref” for the reference mixture. The values for |S_21_|_sam_ and |S_21_|_ref_ in the matrix are determined as *f*_sam_ and *f*_ref_, respectively.

However, in previous analyzing the measuring of the complex permittivity was decided relying on the insertion loss and conductivity parameters, hence the matrix in Equation (3) will be:

(4) $$\left[\begin{array}{c} \Delta \sigma \\ \Delta \left|S_{21}\right| \end{array}\right]=\left[\begin{array}{cc} m_{11} & m_{12}\\ m_{21} & m_{22} \end{array}\right]\cdot \left[\begin{array}{c} {\Delta \varepsilon \prime }_{\mathrm{sam}}\\ {\Delta \varepsilon \prime \prime }_{\mathrm{sam}} \end{array}\right]$$

where Δσ_sam_ = Δσ_sam_ − Δσ_ref_

From data of Figure 16d, we have obtained the calibration curve for between conductivity and insertion loss, using linear regression. Such a curve is

(5) $$\left[\Delta \sigma \right]=\left[0.0985\right]\cdot \left[\Delta \left|S_{21}\right|\right]$$

For Equation (4), the relative resonant conductivity, Δσ and relative magnitude of parameter *S*_21_, Δ$\left|S_{21}\right|$ are computed and given in Table 4. The reference for both the conductivity and the transmission coefficient is the sample containing 100% water.

The parameters *m*_11_, *m*_12_, *m*_21_, and m_22_ are related to the electrical characteristics of the fabricated sensor. According to Equation (4), a set of linear functions used for describing the relationship of resonance characteristics of the sensor and the complex permittivity of the liquid sample under test can be accurately stated as follows [26]:

(6) $$\left[\begin{array}{c} \Delta \sigma \\ \Delta \left|S_{21}\right| \end{array}\right]=\left[\begin{array}{cc} 0.0507 & 0.0338\\ 1.6464 & -6.8621 \end{array}\right]\cdot \left[\begin{array}{c} {\Delta \varepsilon \prime }_{\mathrm{sam}}\\ {\Delta \varepsilon \prime \prime }_{\mathrm{sam}} \end{array}\right]$$

By inverting the matrix in Equation (6), a mathematical model for determining the complex permittivity of unknown liquid sample is derived as:

(7) $$\left[\begin{array}{c} {\Delta \varepsilon \prime }_{\mathrm{sam}}\\ {\Delta \varepsilon \prime \prime }_{\mathrm{sam}} \end{array}\right]=\left[\begin{array}{cc} 17.0041 & 0.0838\\ 4.0797 & -001256 \end{array}\right]\cdot \left[\begin{array}{c} \Delta \sigma \\ \Delta \left|S_{21}\right| \end{array}\right]$$

**Figure 21:**
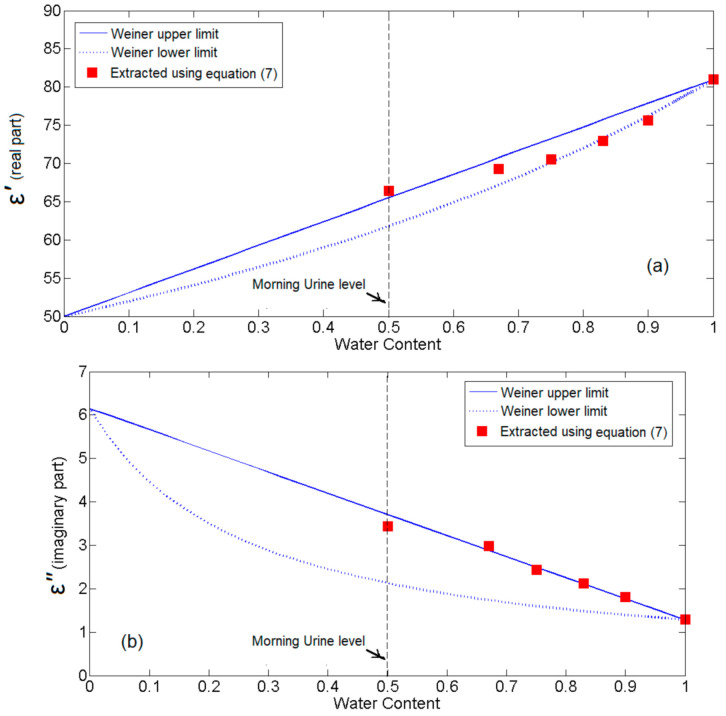
Both the real (**a**) and the imaginary (**b**) parts of the permittivity in mixtures of urine/water.

Using Equation (7) and the data (Δσ and $\Delta \left|S_{21}\right|$) of Table 4, we have obtained the real and imaginary part of the complex permittivity for the different mixtures of water in urine (Figure 21). Indeed, urine–water mixture is not a binary mixture such as ethanol–water, methanol–water, etc. Nevertheless, a good assent with the forecast presented by the Weiner model that extracted relying on simulation results (the upper and lower limits of that model are also specified in Figure 21) [27]. Evidence the validity of the proposed sensor to determine the reasonable complex permittivity of urine-water mixture. The computed values of real and imaginary parts are added to Table 4.

For comparison objectives, the static Weiner model is also included. Next, we calculate the sensitivity of the sensor. The sensitivity in resonance-based microfluidic dielectric sensors is defined as [26]:

(8) $$S\left(\%\right)=\frac{f_{\varepsilon_{r}}-f_{ref}}{f_{ref}\cdot \left(\varepsilon_{r}^{\prime }-1\right)}$$

where $f_{\varepsilon_{r}}$ represents the resonant frequency of the urine–water mixture, $f_{ref}$ represents the resonant frequency of the unloaded sensor and $\varepsilon_{r}^{\prime }$ represents the real part of the relative permittivity.

Using Relation (8) and the data from the measurements, the sensitivity of the proposed sensor for organic fluids is determined. Based on different samples, different values for the sensitivity are obtained, as shown in Table 4.

The results from Table 4 show that the sensor can be used successfully to characterize organic liquid mixtures by their electrical properties. Additionally, the sensitivity has good results.

## 4. Discussion

In Table 5, the results for the sensitivity are compared to the ones in the literature. Note that there few investigations regarding measurements of mixtures between water and organic liquids, such as urine. Still, the references in Table 5 refer to sensors measuring liquid mixtures with high relative permittivity.

Interpreting the results obtained in comparison to other sensors, it can be observed that the proposed sensor offers the best sensitivity. Nevertheless, it is used to electrically characterize samples containing organic liquids, such as urine. Urine is a complex compound made of inorganic and organic compounds. All of them influence the electrical permittivity, so it is very important to have accurate sensors to determine these parameters.

When compared to other resonance-based microwave microfluidic sensors, it can be observed that the relative permittivity is in a narrower range, from 66 to 74.

Additionally, the proposed sensor is not a microfluidic one but still has some advantages when compared to them. The main advantages refer to the fact that the technological process is much simplified as no microfluidic channels are required, just a Teflon beaker attached to the sensor, which does not influence the frequency behavior of the sensor. Additionally, the sensing area is in a better contact to the beaker due to its flat shape rather than microfluidic channels which have a cylindrical shape and thus, limiting their contractability to the flat surface of the sensor. Nevertheless, no mechanical systems for pumping the liquid in the capillaries is required and no synchronization between filling the microfluidic channels with the LUT and reference liquid is needed. Another advantage is that it is implemented on an affordable substrate, such as FR-4. Urine can be provided in large quantities, while the capacity of the beaker is less than 1 mL.

In the literature, many studies refer to sensors used to characterize inorganic samples rather than organic ones. The sensor in [31] is used to characterize the samples of urine only from the conductivity perspective and a color chart. No information about the complex permittivity is given.

In [35] the urine samples are characterized by means of concentrations of electrolytes. The sensor proposed in this paper can detect electrolyte concentrations as small as 0.25 g/L, with maximum sensitivity of 0.033 (g/L) − 1. The sensor is validated by measuring the concentration of three types of electrolytes, i.e., NaCl, KCl, and CaCl_2_ from urine. Again, the complex permittivity is not given.

Thus, the sensor proposed in this paper can be used successfully to detect with great sensitivity the changes in the values of the complex permittivity of urine samples. This can be used to determine metabolic changes and help diagnose different disorders.

## 5. Conclusions

A highly sensitive differential microstrip sensor for biomedical sensing applications is designed, fabricated, and tested. It consists of two identical parts, each of them made of a Wilkinson power divider and a transmission line loaded with two open-stub resonators. The structure is easily fabricated on a single metal microstrip layer. The Teflon beaker is placed on top of the microstrip surface instead of having a microfluid channel etched, thus simplifying the production process. The samples used for measurements were a mixture between water and urine with different percentages. The results were used to determine the complex permittivity of the liquid mixtures, including pure water and pure urine. Due to starting with different mixture sample that are supplied in the simulation, the data range of water content that was used in the simulation was recalibrated to match the same data range of the measured samples. As the result, good assent between the measured complex permittivity values and that forecasted by the Weiner model that extracted relying on simulation results. The values for the complex permittivity show good agreement with reference values. Additionally, the sensitivity of the sensor determined based on measurements is very good in comparison with similar works. The sensor can successfully be used in medical applications that require investigating the electrical parameters of urine in different medical conditions.

## Figures and Tables

**Table 1:** The electrical and physical parameters of the Wilkinson power divider.

Name of the Parameter	Value of the Parameter
Reference impedance (*Z*_0_)	50 Ω
Central frequency (*f*)	1.245 GHz
Resistance (*R*)	100 Ω
Width of the access transmission line (*W*)	3.083 mm
Width of the impedance inverter transmission line (*w*)	1.606 mm
Length of the impedance inverter transmission lines (*ℓ*)	75 mm

**Table 2:** Urine–water mixture samples.

Sample	Water (%)	Urine (%)
1	100	0
2	80	20
3	66	33
4	50	50
5	33	66
6	0	100

**Table 3:** Urine-water mixture samples and their calibrated results.

Sample	Water (%)	Urine (%)	Water (%) after Calibration	Morning Urine Calibration (%)
1	100	0	100	0
2	80	20	90	10
3	66	33	83	17
4	50	50	75	25
5	33	66	67	33
6	0	100	50	50

**Table 4:** Measurement results.

Sample	S_21_ (dB)	Quality Factor	Δσ (S/m)	$\Delta \left|S_{21}\right|$ (dB)	ε′_r_	ε″_r_	S (%)
1	−26.93	34	-	-			
2	−23.39	23.181	0.348	3.54	75.6161	1.8058	2.53
3	−21.87	21.659	0.4986	5.06	72.9279	2.1292	2.71
4	−21.1	20.632	0.5744	5.83	69.3282	2.4418	2.84
5	−20.96	16.839	0.5882	5.97	69.3282	2.98054	2.826
6	−19.32	14.1456	0.7498	7.61	66.4429	3.4337	2.89

**Table 5:** Comparison between various resonance-based microwave microfluidic sensors.

Sensor	Type of Fluid	Central Frequency (GHz)	ε′_r_ Range	S (%)	References
Substrate integrated waveguide	Isopropanol	3.6	4–76	0.15	[28]
CSRR	Ethanol	2.37	9–79	0.03	[29]
Shunt-connected series LC resonator	Ethanol	2	30–80	0.44	[26]
CSRR	Ethanol	1.6	30–80	0.626	[19]
Open CSRR	Methanol	0.9	35–80	1.8	[30]
CSRR	Urine	4	-	-	[31]
Dumbbell-Shaped Defect Ground Structures	Isopropanol	1.05	75–80	1.02	[32]
CSRR	Ethanol	1.618	9–79	0.626	[18]
RLC	Glycerol	2.3	8.22–79.5	2.117	[33]
Open SRR	Isopropanol	1.8	75–80	1.6	[34]
Classic	Glycerol/Ethanol	1.9	-	1.316	[35]
Open stub resonator	Urine	1.25	66–74	2.53	Proposed
